# Supplementary material for: Clinical Application of Epithelial Sodium Channel (ENaC) as a Biomarker for Arterial Hypertension
Source: Biosensors (Basel). 2022 Sep 29;12(10):806. doi: 10.3390/bios12100806 (PMC9599886; doi:10.3390/bios12100806)
Supplement: Supplementary file 1 [file biosensors-12-00806-s001.zip › biosensors-1862578-supplementary.pdf]

Supplemental material

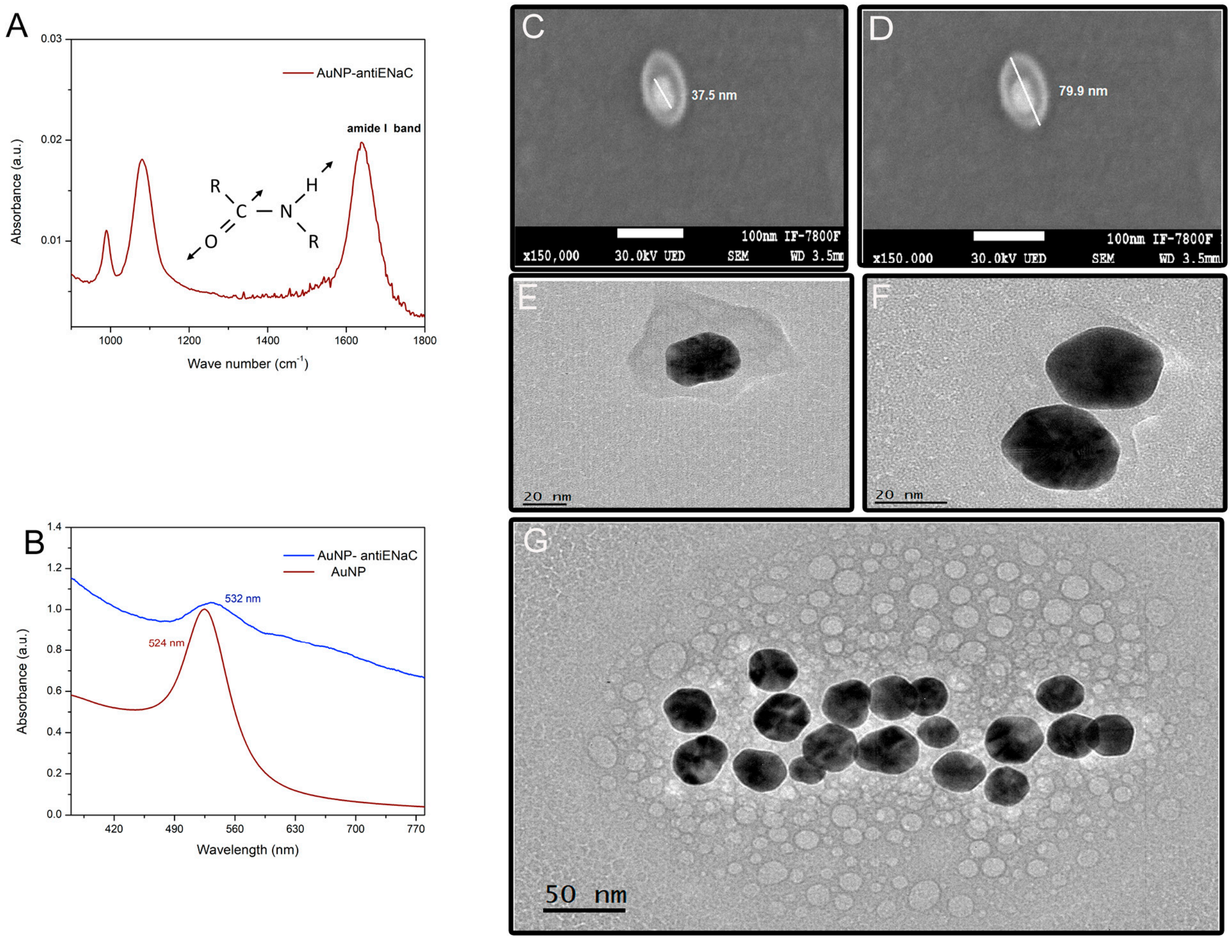

Figure S1. Physicochemical characteristics of the AuNP-anti-ENaC bioconjugates. AuNPs covered with BSA-antibody complexes: (A) FTIR-ATR absorbance spectra of BSA and AuNP-anti-ENaC bioconjugates, (B) UV–VIS spectra of AuNPs, AuNPs functionalized with MPA, and AuNP-anti-ENaC bioconjugates, (C,D) AuNPs observed by scanning electron microscopy, (E–G) AuNPs observed by transmission electron micrographs.

Table S1. Measurements carried out in water in Zetasizer Nano-ZS90 equipment; the measurement of each sample was repeated three times.

AuNPs-anti-ENaC + BSA concentrations[mg/mL]	Size (nm) ± SD	PdI	Zeta pot (mV) ± SD
0.0025	213 ± 5	> 0.6	-5.7 ± 1.03
0.005	286 ± 9	> 0.6	-18.8 ± 5.4
0.01	261± 3	> 0.6	-27.8 ± 7.3
0.1	202 ± 7	0.4	-27.3 ± 1.3
0.2	178 ± 8	0.6	-29.7 ± 1.8
0.3	162 ± 2	0.6	-31.3 ± 3.4
0.6	165 ± 7	0.5	-37.2 ± 6.7
1.0	169 ± 17	0.5	-31.4 ± 3.6
